# The Influence of Environmental Conditions and Husbandry Practices on Goat Welfare

**DOI:** 10.3390/ani16050838

**Published:** 2026-03-07

**Authors:** Renata Pilarczyk, Małgorzata Bąkowska, Agnieszka Tomza-Marciniak, Jan Udała, Beata Seremak, Ewa Kwita, Piotr Sablik, Bogumiła Pilarczyk

**Affiliations:** 1 Department of Biostatistics, Bioinformatics and Animal Research Methods, Faculty of Biotechnology and Animal Sciences, West Pomeranian University of Technology in Szczecin, Klemensa Janickiego 29, 71-270 Szczecin, Poland; renata.pilarczyk@zut.edu.pl (R.P.); piotr.sablik@zut.edu.pl (P.S.); 2 Department of Animal Reproduction Biotechnology and Environmental Hygiene, Faculty of Biotechnology and Animal Sciences, West Pomeranian University of Technology in Szczecin, Klemensa Janickiego 29, 71-270 Szczecin, Poland; malgorzata.bakowska@zut.edu.pl (M.B.); jan.udala@zut.edu.pl (J.U.); beata.seremak@zut.edu.pl (B.S.); ewa.kwita@zut.edu.pl (E.K.); bogumila.pilarczyk@zut.edu.pl (B.P.)

**Keywords:** behaviour, environmental enrichment, goat welfare, rearing environment, rearing practices, stress, welfare assessment, welfare assessment protocols

## Abstract

Goat (*Capra hircus*) welfare is an important issue in any farming system. The aim of the study was a comprehensive analysis of the impact of environmental factors and farming practices on the welfare of goats, with particular attention to physical, behavioural, and emotional aspects. It includes a review of the up-to-date literature on the effects of environmental conditions including air temperature, air humidity, space, feeding systems, social relationships (mother–offspring, human–animal, animal–animal), zootechnical procedures (dehorning, castration, hoof trimming) and welfare assessment methods. It compares the AWIN, Anzuino, Muri and Leite protocols for assessing goat welfare and their application in the Five Domain Model. Goat welfare is strongly influenced by their environment, nutrition and socialisation: heat stress and confined space cause physiological disorders, decreased immunity and increased aggressive behaviour and a monotonous diet leads to frustration and reduced cognitive activity, whereas positive early contact with humans reduces anxiety and maintaining the mother–kid bond supports the social development of young goats. Furthermore, significant improvements in welfare and stress reduction can be achieved by providing anaesthesia and painkillers where necessary to minimise pain and enriching the environment with items that support natural behaviour, such as platforms, brushes and items for cognitive tasks. In general, the keeper should take a holistic approach, combining environmental optimisation, humane husbandry practices and regular monitoring using validated assessment protocols to improve welfare. These measures are both an ethical obligation and a prerequisite for animal health and production efficiency. Nevertheless, there is a need for further research focussing on the development of non-invasive assessment methods and innovative forms of environmental enrichment.

## Simple Summary

Goat welfare is an important issue in any farming system. This paper presents a comprehensive analysis of the influence of environmental factors and husbandry practices on goat welfare, with a particular emphasis on physical, behavioural and emotional aspects. It includes a review of the up-to-date literature on the effects of environmental conditions including air temperature, air humidity, space, feeding systems, social relationships (mother–offspring, human–animal, animal–animal), zootechnical procedures (dehorning, castration, hoof trimming) and welfare assessment methods. It compares the AWIN, Anzuino, Muri and Leite protocols for assessing goat welfare and their application in the Five Domain Model. Goat welfare is strongly influenced by their environment, nutrition and socialisation: heat stress and confined space cause physiological disorders, decreased immunity and increased aggressive behaviour and a monotonous diet leads to frustration and reduced cognitive activity, whereas positive early contact with humans reduces anxiety and maintaining the mother–kid bond supports the social development of young goats. Significant improvements in welfare and stress reduction can be achieved by providing anaesthesia and painkillers where necessary to minimise pain and enriching the environment with items that support natural behaviour, such as platforms, brushes and items for cognitive tasks. In general, the keeper should take a holistic approach, combining environmental optimisation, humane husbandry practices and regular monitoring using validated assessment protocols to improve welfare. These measures are both an ethical obligation and a prerequisite for animal health and production efficiency.

## 1. Introduction

Modern approaches to food production increasingly emphasise the role of animal welfare, and the rearing conditions of livestock are under increasing scrutiny by both consumers and producers. An important role in this is played by the European Green Deal, which promotes sustainable agriculture and measures aimed at protecting the environment and improving farming conditions.

As a result of this increasing environmental awareness, goat farming has grown in popularity as an ecological alternative to other animal production systems, which has brought with it an increasing need to monitor their welfare. As with other animals, to ensure goat welfare, it is necessary to both minimise suffering and stress and promote positive experiences so that positive emotional states outweigh negative ones [1,2]. As such, the animals should be allowed to maintain their physical health, experience positive emotions and express species-specific behaviours [3,4].

On livestock farms, goats often live in large herds, in high densities or in groups composed of unrelated individuals. Such environments differ significantly from their natural habitat, and recognising other members of the group and maintaining lasting social bonds can pose a significant challenge [5,6,7]. Stanley and Dunbar [7] observed that, for cognitive and behavioural reasons, the optimal group size is 12 or 13 individuals. Frequent regroupings, typical of intensive livestock systems, can therefore have a direct and strong impact on the social dynamics within the herd, leading to the need to reestablish relationships and hierarchies. However, it should be emphasised that the stability of the social bond is linked to the ability to effectively recognise other individuals within the herd. If group composition changes frequently or the group is too large, these mechanisms cannot be fully utilised, which promotes social instability and increases stress, especially for individuals with lower social status [5,6,7].

Goat welfare is strongly influenced by feed type and presentation. Indeed, Cellier et al. [8] note that goats prefer leaves to grass, and their feeding behaviour appears to be influenced by the height and type of feeder. Introducing greater variety in feed delivery has also been found to allow the goat to better express its natural feeding patterns and supports cognitive activity.

Due to their complex behavioural needs and cognitive abilities, goats may be particularly sensitive to the constraints of intensive farming systems [5,9]. As such, there is a need to analyse goat welfare to provide an understanding of how husbandry conditions affect the health, emotions and natural behaviours of the animal. The results enable more accurate identification of problems deriving from of the intensive farming system. They can also foster the development of strategies to improve husbandry, including those associated with environmental enrichment, which can reduce stress, increase positive experiences and better align their behaviour with their natural needs.

Welfare encompasses both the physical and mental state of an animal; therefore, its assessment cannot rely on a single parameter. The starting point for assessing goat welfare is the selection of appropriate indicators that allow for the reliable and repeatable determination of specific parameters reflecting the animals’ ability to cope with particular housing conditions [10,11]. For a welfare indicator to be considered valid, it must meet several criteria: it should be biologically accurate, sensitive to changes in conditions, reliable (providing consistent results regardless of the observer—both intra- and inter-observer reliability—and the timing of measurement, i.e., objective and repeatable), and feasible in farm practice (considering time, cost, and technical requirements) [12,13,14]. In practice, both animal-based and resource-based indicators are used. Animal-based indicators relate directly to the condition of goats and include, among others, lameness, injuries, disease symptoms, and behaviour, thus best reflecting the actual level of welfare. Resource-based indicators, on the other hand, concern environmental and management conditions such as access to feed and water, living space, and substrate quality. Both groups of indicators have limitations: animal-based indicators can be time-consuming and subject to observer bias, whereas resource-based indicators do not account for individual differences and adaptability among goats [15,16,17,18,19]. Protocols for assessing goat welfare developed in recent years are intended for dairy goats kept in intensive systems (AWIN, ANZUINO, Muri). No validated protocols are currently available for meat goats or young goats kept in systems other than intensive ones [11].

The aim of this study is to provide an overview of the impact of housing conditions on goat welfare and their ability to express natural behaviours, as well as to compare the AWIN, Anzuino, Muri, and Leite protocols used for the assessment of goat welfare.

It examines both the physical and emotional aspects of welfare, with a particular emphasis on the effect of the environment on encouraging exploration, social interactions and the expression of species-specific behaviours. Its findings indicate ways in which welfare can be improved by appropriately enriching housing conditions to reduce stress, promoting positive experiences and enabling the expression of natural behaviours, even in intensive farming systems.

## 2. Literature Review Methodology

A corpus of articles was compiled by searching the PubMed, Web of Science, and Google Scholar databases as well as Scopus. The search used the following key words: “goat welfare” OR “*Capra hircus* behaviour” OR “enrichment for goats” OR “environmental enrichment” OR “cognitive enrichment” OR “goat-human interaction” OR “goat social behaviour” OR “social hierarchy in goats” OR “goat stress indicators” OR “handling stress in goats” OR “aberrant behaviour in goats” OR “thermoregulation in goats” OR “behavioural indicators of welfare in goats” OR “welfare indicators” OR “ethological needs of goats” OR “trust” OR “tameness” OR “pain assessment in goats” OR “parasite infestations in goats” OR “congenital and genetic defects in goats” OR “limb deformities in goats” OR “transport stress in goats” OR “exhibition stress in goats” OR “veterinary training” OR “off-label drug use” OR “biosecurity” OR “transport management” OR “quarantine conditions” OR “health monitoring in goats” OR “disease management” OR “animal handling” OR “stress physiology in goats” OR “feeding behaviour in goats” OR “housing conditions” OR “goat reproduction” OR “genetic selection in goats” OR “welfare assessment protocols”.

To narrow down the results, the logical operator AND was used in combination with the word *goat* in some queries. The syntax of the queries was adapted to the search capabilities of individual databases. The search covered primary materials, reviews, books, and scientific reports.

The search of the PubMed, Web of Science, Scopus and Google Scholar databases yielded a total of 1962 publications. After removing duplicates and rejecting less valuable materials from Google Scholar, 1550 unique records remained. A preliminary selection based on titles and abstracts narrowed the collection down to 527 publications. A further evaluation of the full texts allowed us to exclude 85 articles due to lack of access and 312 due to failure to meet the substantive criteria. Ultimately, 130 publications were selected for detailed analysis, which form the basis of this literature review.

The publications were divided thematically into the following areas:

- The Effect of the Husbandry Environment on Goat Welfare;
- Relationships Between Mother and Offspring;
- Relationships Between Goats;
- Human–Goat Relations;
- Goat Welfare During Grooming Procedures;
- Specific Welfare Indicators for Goats;
- Protocols for Assessing Welfare in Goats.

The following inclusion criteria were used for publications:

- Peer-reviewed original research articles, reviews and book chapters;
- Full-text publications allowing for a thorough analysis of research methodologies, results, and standards.

To allow for a comprehensive discussion of the impact of environmental factors and husbandry practices on goat welfare, the collected information was analysed, categorised and organised by topic. The results are presented in the relevant sections.

## 3. The Effect of the Husbandry Environment on Goat Welfare

When kept indoors, goat welfare is influenced by numerous environmental and technical factors.

### 3.1. Air Temperature

Although goats are considered well adapted to tropical climates, their exposure to high air temperatures limits their physiological functions and welfare [20]. The optimal air temperature for goats ranges from 10 to 18 °C (Table 1).

High temperatures reduce the effectiveness of the innate immunity responsible for the early response to pathogens; a consequent decrease in IgM and IgG antibody production also increases the risk of parasitic infection [21,22]. As a result, these animals experience an overall deterioration in health, which is one of the most important indicators of physiological and behavioural well-being. As such, to maintain their welfare, it is important to provide goats with appropriate environmental conditions that minimise heat stress and support natural immune mechanisms.

Prolonged exposure to high temperatures also disrupts their water and electrolyte balance, weakening overall physical condition [23,24,25] and limiting their ability to maintain homeostasis. When kept at high ambient temperatures, goats undergo a number of physiological changes aimed at maintaining a stable body temperature. For example, one of the first symptoms of heat stress is an increase in breathing rate, which allows excess heat to be dissipated through evaporation of water from the respiratory tract [26]. This may be accompanied by an increase in heart rate and blood flow through the vessels of the skin, which allows heat to dissipate more quickly into the environment [23,27,28]. This dilation of blood vessels, and the consequent increase in surface temperature [29], is accompanied by activation of the sweating mechanism, which helps to cool the body through evaporation. However, this process is limited in dehydrated animals, indicating adaptive water management under unfavourable thermal conditions [30,31].

At low temperatures, goats require additional energy for heat, which they acquire through increased feed intake. This is an important consideration for kids, which are particularly sensitive to hypothermia in the first days of life [32]. A study by Sutherland et al. [33] showed that kids preferred to lie in the area where heat lamps were present, regardless of the floor type. These findings indicate that providing an additional heat source (heat lamps) improves their welfare and productivity.

### 3.2. Air Humidity

Air humidity also affects comfort and respiratory health. Excessive levels of water vapour can increase the risk of respiratory disease, especially when combined with elevated temperatures. Therefore, goat housing facilities should limit sources of moisture and ensure its drainage through efficient ventilation to avoid a humid microclimate [32].

### 3.3. Ventilation

The air exchange in the building also plays a very important role in maintaining the health of goats. Well-designed ventilation removes excess moisture, harmful gas admixtures and microorganisms while not creating draughts, to which goats are particularly sensitive. Adequate air flow reduces the risk of mucous membrane irritation and respiratory diseases and improves the overall microclimate [32].

### 3.4. Lighting

Light is important for both goats and workers. Natural or artificial light affects the activity levels, circadian rhythm and well-being of the animals, and good lighting improves the working conditions for the keeper, making it easier to observe the herd and perform daily tasks [32]. Moreover, goats are seasonal short-day breeders, with peak breeding activity occurring in autumn as day length decreases. Their reproductive behaviour and lactation performance are influenced by daylight intensity and the light–dark ratio. However, there are currently no official husbandry regulations regarding lighting conditions in goat housing facilities [34]. Sevi et al. [35] reported that the recommended light intensity in goat housing should be at least 100 lx for a minimum of 8 h per day (Table 1). However, considering the available scientific data, we believe that an 8 h photoperiod may be too short. It was found that an elongated photoperiod positively affects milk yield and body weight gain of kids. For example, Flores et al. [36] observed a higher mean milk yield in goats exposed to a 16 h photoperiod compared to goats exposed to an 11 h photoperiod. In another study, Flores et al. [37] compared body weight gain in kids exposed to a natural photoperiod and a 16 h photoperiod and found that weight gain was significantly higher in the group exposed to the extended photoperiod (*p* < 0.05).

Unfortunately, there are limited data and recommendations regarding light intensity. In earlier studies, recommendations were expressed as glazing-to-floor-area ratio. Accordingly, Toussaint [32] suggested a ratio of 1/20, whereas Sevi et al. [35] suggested a ratio greater than 1/15.

**Table 1:** Recommended environmental parameters for dairy goats in livestock buildings.

Parameter	Minimal	Optimal	Maximum	Notes	Impact on Well-Being/Consequences of Deficiency or Excess	References
Air temperature	6 °C	10–18 °C	27 °C	Goats maintain a constant body temperature of approximately 38.5 °C	<6 °C: hypothermia, increased feed intake; >27 °C: heat stress, reduced feed intake, rapid breathing.	[32]
Relative humidity	60%	70%	80%	Excessive humidity promotes respiratory diseases	Excessive levels: risk of pneumonia; insufficient levels: dry mucous membranes, discomfort.	[32,35]
Ventilation rate	6 m^3^/h (winter)	Summer: - Young animals: 35 m^3^/h - Adult: 70 m^3^/h Winter: - Young animals: 20 m^3^/h - Adult: 45 m^3^/h	120 m^3^/h (summer)	–	Poor ventilation: accumulation of moisture and gases, respiratory diseases, animal stress.	[32,35]
Air flow speed	–	–	Adult goat: 0.5 m/s Kid: 0.2 m/s	–	Airflow speeds above the maximum values: hypothermia, decreased immunity, poor weight gain, respiratory diseases.	[35]
Air volume per animal	–	9 m^3^	–	–	Air volume < 9 m^3^: increased temperature and humidity, increased risk of disease.	[32]
Lighting	–	>100 lx/8 h/day	–	–	Insufficient indoor lighting: biological rhythm disturbances, decreased activity and comfort.	[35]
Concentration of harmful gas admixtures	–	–	CO_2_: <2500 ppm NH_3_: <10 ppm H_2_S: <2.5 ppm	Higher concentrations are harmful to the respiratory system	Exceeding standards: irritation, lung disease, decreased productivity.	[35]
Area available per adult goat (in stall housing)	0.5 m^2^	–	–	–	<0.5 m^2^: stress, aggression, restricted movement, increased risk of disease.	[32]
Area available per adult goat (in a pen, separately)	1.5 m^2^	–	–	–	Reduction to 1 m^2^: stress, restricted movement.	[25,37]
Area available per adult goat (in a pen, in group)	1.5–2 m^2^	–	–	–	Reduction to 1 m^2^: stress, aggression, restricted movement, reduction in feeding activity, shorter resting time, increased risk of disease.	[34]
Area available per adult goat in open housing with an outside yard	1.5 m^2^	–	–	–		[32]
Additional area available per kid before weaning	0.2–0.3 m^2^	–	–	–	<0.2 m^2^: stress, impaired motor development, increased mortality.	[32]
Area available per kid after weaning	0.2–0.4 m^2^	–	–	During the first 30 days	Area below minimum resulted in restricted movement, stress, higher risk of illness.	[32,38]
Area available per adult goat–ram	2.2–2.5 m^2^	–	–	–	Insufficient space: stress, aggression, restricted movement.	[35]
Length of space at drinking troughs (after weaning)	–	0.33 m	–	At drinking troughs/gutters	Insufficient space causes stress and limits water intake.	[32]
Feeding area	0.4 m per goat	–	–	–	Insufficient space causes competition, stress, feed shortage.	[32]
	0.2 m per goat	–	–	–		[35]

### 3.5. Stocking Density

The amount of area available to the goats influences their resting patterns. Andersen and Bøe [39] report that, when the resting area per goat falls below 1 m^2^, fewer animals lie down in the designated area at the same time, and some goats move to activity zones. Loretz et al. [40] observed a similar effect: reducing the lying area from 2 m^2^ and 1.5 m^2^ to 1 m^2^ per animal also decreased resting and feeding time. This space limitation had a stronger effect on individuals lower in the hierarchy, which spent more time in less comfortable activity areas than the more dominant animals.

When resting, goats favour locations next to the walls and tend to avoid the centre of the pen; this behaviour is considered instinctive and may offer a sense of security against potential predators [15].

Access to an outdoor run may positively influence goats’ activity and social behaviour and thereby contribute to improved welfare [41]. Studies have shown that, when given such access, goats spent a large part of their time more actively with a reduction in resting time. This was influenced by both the larger area available to the animals and the varied environment, e.g., the presence of branches for chewing and exploration [38]. Negretti et al. [42] observed that goats with access to outdoor areas were more active in socialisation and show a greater disposition to perform their natural behaviour patterns.

### 3.6. Flooring and Bedding

The welfare of goats, particularly in intensive husbandry systems, is strongly influenced by the type of flooring and bedding implemented, which directly impacts physical comfort, environmental hygiene, and hoof integrity (Table 2). Organic bedding materials, such as straw and wood shavings, provide both thermal insulation and mechanical cushioning, thereby facilitating resting behaviour and reducing biomechanical stress on the musculoskeletal system. However, the positive effects of such substrates on welfare are highly dependent on maintaining a dry and hygienic environment, as elevated moisture levels promote microbial proliferation, predisposing animals to hoof pathologies (e.g., foot rot, dermatitis) and other environmentally mediated infections [32,33,43,44].

Goats are most often kept on deep bedding (straw), which plays an important role in ensuring comfort during rest [45]. It is important that the bedding material is both soft and thick, as well as dry and free from faeces or other dirt. A bedding layer that is too thin, wet or dirty reduces comfort when lying down, which can negatively affect well-being [17]. In studies investigating goats’ bedding preferences, Ref. [43] demonstrated that goats most commonly selected straw bedding, followed by rubber mats and solid wooden floors. In areas covered with wood shavings, Sutherland et al. [33] observed a higher frequency of urination and defecation by goats, indicating that this substrate is preferentially used for elimination rather than resting.

The state of bedding should be assessed visually within the pen: the floor should be completely covered with material and its quantity and quality should ensure comfortable rest. Studies show that even a limited amount of straw can provide adequate lying comfort for goats without adversely affecting milk hygiene or the bedding itself [44]. Straw bedding has been shown to be beneficial for goat welfare, increasing lying time, reducing aggression and promoting grooming behaviour [46].

**Table 2:** Comparison of bedding types used in goat husbandry and their impact on animal welfare.

Type of Flooring/Bedding	Advantage	Disadvantages /Limitations	Impact on Welfare	References
Straw	Comfortable, insulating, moisture-absorbing	Requires regular replacement, retains moisture, and creates a favourable environment for bacterial growth.	Promotes resting behaviour and reduces musculoskeletal stress, but excessive moisture can increase risk of hoof diseases and infections.	[33,43]
Wood shavings	Moisture-absorbing, maintenance of hygiene	May clump together and promote bacterial growth if too wet.	Helps maintain a dry surface, reducing the risk of hoof diseases and improving comfort and hygiene; when other types of flooring are available, goats tend to use it as an eliminative surface (a place for defecation).	[33]
Rubber mat/flexible mat	Easy to clean and disinfect, reduces pressure on the hooves, preferred on hard floors	Expensive; requires regular removal of manure.	Provides lying comfort, cushioning, and thermal insulation; when other flooring types are available, it is most often chosen by goats for resting.	[47]
Solid floor: concrete or metal floor	Durable, easy to clean and disinfect	Hard and cold; requires regular manure removal and may cause hoof problems.	Can negatively affect resting comfort and hoof health; requires additional bedding to mitigate welfare risks.	[47]

Inorganic bedding, particularly rubber mats, provides a stable and resilient surface that reduces pressure on the limbs and allows animals to adopt natural lying postures. Studies indicate that goats clearly prefer such bedding compared with slatted or metal floors, which are considered the least comfortable. However, the hygienic effectiveness of rubber mats depends on regular removal of excreta [33,39,43,48].

Slatted floors and metal grids offer superior hygiene by effectively removing excreta and reducing moisture and ammonia concentration, thereby decreasing the risk of infections [33,43]. At the same time, their hard and uneven surface limits lying comfort and may lead to injuries and uneven hoof wear [33,45].

Intermediate solutions, such as partial slats or floors with cushioning elements, partially balance these opposing effects by combining improved hygiene with higher animal comfort [48,49].

### 3.7. Foraging

In their natural environment, goats have access to a variety of plant parts, including leaves, shoots and grasses. As such, they are able to select a varied diet and, in doing so, adopt a variety of feeding postures, allowing them to pick food at different heights ranging from the ground to tree branches [50,51]. These feeding behaviours are also influenced by their food preferences: goats are naturally leaf-eating animals and prefer leaves to grass. They also enjoy exploring their surroundings, and engaging in behavioural activities in line with their natural circadian and seasonal rhythms promotes both physical and mental well-being [8] (Table 3).

In extensive husbandry systems, goats have limited but partially preserved access to natural food and space, allowing them to choose from the available vegetation, adopt some natural feeding postures and maintain a moderate ability to explore [52]. As a result, they retain most of their natural behaviours and feeding preferences, which, again, supports their behavioural and cognitive well-being (Table 2).

In intensive systems, goats have limited food choices, being mainly fed silage and concentrated feed in troughs (Table 2). Under such conditions, their natural feeding behaviours and cognitive behaviours are restricted, and they have less opportunity to explore their environment [52,53,54]. Furthermore, the use of artificial feeding times and limited space can lead to reduced motivation, boredom, frustration, and stress and goats can show signs of undesirable behaviours such as chewing on elements of the pen [8,55].

By providing goats access to different types of feed and offering them the ability to choose the height of the trough, they are allowed to engage in more natural behaviours that require the use of their cognitive abilities [8].

### 3.8. Kneeling at the Feeding Rack

Feeder design can also affect comfort, behaviour and stress in goats. Improperly designed feeders, e.g., those that are too low or restrict access to feed, increase agonistic behaviour and stress, as measured by heart rate variability and cortisol levels [56]. In such cases, the goat may adopt a kneeling position at the feeder, resting on its hind legs and wrist joints, and lifting the rear part of the body [14,17]. Therefore, recording the number of goats assuming this posture can be a practical way for identifying difficulties in using feeders, resulting in potential joint and muscle overload. This can lead to reduced feed intake and decreased condition and performance.

### 3.9. Queuing at Feeding

In most husbandry systems, goats will be forced to wait in line for other individuals to finish feeding before taking their turn. By observing this behaviour, it is possible to determine whether all animals have equal access to food and whether there are enough places at the feeder. When not all goats can eat at the same time, this can lead to stress and affect interactions within the herd, especially in groups with hierarchical positions or in mixed herds [17]. Prolonged waiting can also influence social behaviour, such as aggression between goats or withdrawal by lower-status animals [12,57].

The assessment itself involves systematically recording the number of animals in the queue at specific intervals, beginning a few minutes after providing feed. The measurements can be taken from outside the pen, i.e., from a location that provides a good view of the entire trough. A goat is considered to be waiting in line when it stands close to another animal feeding and is clearly waiting for its turn [13,17]. This indicator is easy to observe in farm conditions and can be used to detect problems with access to food [12,13,57].

### 3.10. Queuing at the Drinking Trough

Another important indicator of goat welfare is the queue at the drinking trough. Studies confirm that increasing the number of animals per drinking trough leads to increased competition for water, with the greatest number of agonistic interactions and longest queues observed when 30 goats were sharing each trough [58]. Queues occur when goats have to wait for other animals to finish drinking. These data can indicate whether all individuals have equal access to water and whether sufficient drinking places have been provided [17].

The assessment is carried out outside the pen from a location that can provide a view of all functioning troughs. The observation begins when the first goat starts drinking and lasts for approximately 15 min. During this period, any waiting animals are counted, i.e., those standing in line approximately 50 cm behind the drinking goat with their heads turned towards the trough [17]. The absence of a queue indicates adequate access to water, while the appearance of queues may indicate an insufficient number of troughs or social tensions within the group.

A lack of ready access to water is associated with greater stress and can affect social behaviour, especially in animals lower down in the hierarchy [18]. Where the goats are forced to compete for access to water, the resulting stress can cause social tension, provoke aggression between animals and disrupt their natural social behaviour. The presence of such queues is an indicator of competition for water access and the mental well-being of goats. On farms, to reduce stress and potential conflict, it is recommended that there are no more than 15 animals per nipple drinker [58].

### 3.11. Cleanliness

It is important that goats stay clean to ensure their health and well-being. Dry straw bedding reduces the soiling of their coats [15,16].

The AWIN protocol [12,13] specifies that goat cleanliness should be assessed visually by observing their coats and noting the presence of mud, moisture or yellowish discolouration of the coat. However, as the result is significantly influenced by coat colour, as dirt is less visible on dark-coloured goats, this method was abandoned. Instead, the final protocol requires an assessment of the condition and cleanliness of the bedding in the pen. Some researchers have not found coat colour to hinder cleanliness assessment, although this may be due to more accurate and longer observations of individual animals [16,19,59].

The cleanliness of goats is also influenced by their housing conditions, such as regular bedding replacement and clean corridors, or providing access to pasture or outdoor runs [16]. Some authors indicate that soiling around the tail may indicate digestive problems or feeding errors [60].

### 3.12. Enriching the Living Environment of Goats

In their natural environment, goats are able to explore their surroundings and respond to a variety of stimuli, thus allowing them to engage in natural behaviours. This has a positive influence on their welfare [61]. However, such opportunities are limited in intensive farming systems, which reduces activity and prevents positive behaviours such as play, climbing and social interaction. As such, goats kept in such systems are more prone to stress [61,62].

Environmental enrichment can be divided into a number of categories. Physical enrichment includes objects and structures that goats can manipulate or play with, such as brushes, tyres, branches and climbing platforms, and providing access to outdoor runs [63]. Sensory enrichment involves stimulating the senses, for example by playing music or various sounds. Social enrichment focuses on fostering beneficial interactions with humans, which helps to reduce stress and improve animal–carer relationships. Finally, cognitive enrichment requires animals to learn or solve tasks, with food or water as rewards [63].

Goats are naturally curious animals and will eagerly explore new objects and their surroundings [9,55,64,65]. To allow them to exercise this curiosity, and thus fulfil their natural needs, their environment should be enriched by introducing toys, climbing structures, sensory stimuli and devices that extend feeding time, and providing opportunities for positive interactions with humans. Such enrichment has been found to reduce stress and improve welfare [66,67].

Wein et al. [68] give a practical example of the implementation of various forms of enrichment, in which movable brushes were introduced to allow goats to scratch themselves and wooden oak platforms were introduced to serve as resting places and climbing platforms. These structures increased the usable space of the pen, stimulated physical activity, supported social interaction and allowed the goats to fulfil their natural desire for exploration.

The use of physical enrichment has been found to confer measurable behavioural benefits in various environments. In Saanen goat breeding, the presence of climbing trunks, tyres, brushes and corn bottles encouraged the animals to be more active, enabled them to adopt natural postures such as standing on two legs, and reduced stereotypical behaviour [66]. Similar effects were observed in Meriz goats, which actively explored the environmental objects, resulting in a lower frequency of repetitive behaviours [69]. Young dwarf goats benefiting from both structural and cognitive enrichment demonstrated better learning abilities and increased adaptability, indicated by a greater willingness to explore unfamiliar environments [63]. Additionally, Bøe et al. [54] report that providing access to an outdoor enclosure and elements such as branches increased activity, exploration and positive social behaviour in goats.

Various forms of environmental enrichment for goats and their impact on animal behaviour and welfare are presented in Table 4. The table includes physical, sensory, social and cognitive enrichment, along with a description of their effects on animals and references to relevant studies.

## 4. Relationships Between Mother and Offspring

Research shows that kids are able to recognise their mother in the first days of life; they also show a clear preference for staying close to her, which promotes rapid bonding [70]. Contact with their mother reduces stress levels in young goats and allows them to express natural social behaviours that are important for their welfare, such as licking, cuddling and interacting with their siblings [71].

The length and form of contact between mother and kid also have a significant impact on goat welfare. Kids that remain in full round-the-clock contact with their mother exhibited less avoidance behaviour and more safety-related behaviour, such as hiding near their mother in the first weeks of life, compared to those that were partially or completely separated [71]. Maintaining contact allows the young to suckle naturally; this has a beneficial effect not only on their welfare, but also on mammary gland development and milk quality.

The pain experienced during labour affects the behaviour of mothers, especially those giving birth for the first time, which may delay initial contact with their kids. However, the natural need for isolation in a safe place allows for focused care of the offspring and the formation of a strong bond with them [72,73,74]. More difficult births, such as dystocia, may limit maternal interest in the newborn and increase avoidance behaviour, which negatively affects the well-being of both mother and kid [75].

The temperament of the mother plays an important role in their caring behaviour. Those with a calm temperament are more likely to initiate contact with their kids, lick them longer, stay closer to them and respond more quickly to their needs; such behaviour promotes the development of stable bonds and increases the chances of survival for the young [76,77]. Temperament can also influence the reactions demonstrated by the mother at critical moments during birth and in the following hours, and understanding these relationships can support better herd management and kid welfare.

Welfare assessments should include observations of caring behaviour, the non-verbal responses of mothers (e.g., body position, vocalisation intensity) and stress hormone levels, such as cortisol and oestradiol [72,78]. Encouraging greater mother–kid contact and natural suckling enhances the welfare of the young, as well as lactation, milk quality and composition, including its fat and protein content [71].

In addition, long-term observations indicate that kids raised in close contact with their mothers show better social development, lower susceptibility to stress and higher adaptive skills within the group. These characteristics play a key role in maintaining welfare in breeding systems [71].

## 5. Goat–Goat Relations

Goats form social groups that maintain cohesion through their ability to recognise individual conspecifics and through various forms of communication, such as olfactory signals, vocalisations, body postures, and tactile contact [79,80]. Goat social behaviour can be divided into agonistic and affiliative types. Affiliative behaviours, such as grooming, sniffing, and resting in close physical contact, promote social bonding, enhance group cohesion, and reduce the frequency of conflicts [5,39,81]. In contrast, agonistic behaviours—such as head-butting, pushing, biting, threatening postures, and chasing—commonly observed during the establishment and maintenance of herd hierarchy constitute a significant factor in reducing the welfare of subordinate individuals. These behaviours are exacerbated under conditions of limited space, high stocking density, or frequent changes in group composition, leading to restricted access to resources, mechanical injuries, and deterioration of physical condition and health, thereby negatively affecting the expression of natural behaviours and overall welfare [5,39,82,83,84].

## 6. Human–Goat Relations

Human–animal relationships are made up of various mutual contacts and reactions that reflect the level of familiarity, or distance, between the two parties and develop through daily interactions [85]. Farm animals can react to humans in different ways. Some appear fearful and try to avoid contact or appear relatively indifferent, although possibly maintaining a distance. Alternatively, those without fear may engage in positive interactions, allowing touch and close contact [86].

Their perception of humans is influenced by both genetic factors and husbandry practices, such as gradual habituation and early experiences of positive interactions [87]. Those that have experienced higher levels of contact with humans since birth tend to be calmer and more docile and behave more freely around humans than those that have not. In a study of young goats isolated one week after birth, Boivin and Braastad [88] found that those treated gently by humans spent more time in their company than those that were not. Conversely, the goats that were not stroked when young showed less interest in humans and avoided them; this negatively affected welfare and resulted in higher levels of fear, which made it difficult to perform procedures requiring direct contact, such as milking or hoof trimming [89].

Relationships with humans have been found to play a key role in ensuring well-being among animals [90]. This is particularly important in the case of goats, which can often react with fear to humans and are often not accustomed to frequent contact with their caretakers [91,92], especially in extensive farming systems.

Studies indicate that human–animal interactions have a direct impact on the welfare of goats. The influence of humans on herd behaviour appears strongest during milking. In goats, negative relationships with the caretaker, and the associated fear, can lead to reduced milk production and a lower milk letdown [93].

Interestingly, in addition to short-term behavioural responses, the quality of human interaction can also affect the physiological development of animals. Goats that were exposed to regular positive contact early in life were found to achieve greater body weight and heart mass, suggesting that social experiences may have had a long-term impact on the development of stress and metabolic systems [94].

Various studies, conducted under various test conditions on farms, have been performed to assess the reaction of goats to human contact and handling [88,93,95,96]. The research considers both behavioural parameters, such as time spent near humans, number of contacts or vocalisations, and physiological indicators, such as heart rate or milk secretion. Some tests also employ detailed behavioural scales to assess excitability, tension, alertness, anxiety, confidence, friendliness and fear of humans. Their findings provide an insight into how goats react to human presence, movements and attempts at physical contact, as well as everyday activities such as milking. They also make it possible to determine the overall level of comfort, fear and acceptance of humans experienced by goats, which has a significant influence on animal welfare and the safety and efficiency of people working with goats (Table 5).

## 7. Goat Welfare During Grooming Procedures

In dairy farms, young kids often have their horn buds removed in the first days of life to reduce the risk of injury to animals or humans. Adult goats may also be subjected to dehorning or the surgical removal of horns when it was not possible to remove the horns at an early age. Horn removal facilitates herd control, reduces the risk of injury during feeding or transport, and makes it easier to handle the animals [98].

This is a painful procedure in which the horn buds are cauterised with a heated tool. The procedure is also associated with a risk of serious injury, such as brain damage or death; as such, the procedure should be performed by a veterinarian or a person under their supervision [99].

The procedure is very stressful and painful [100]. The animals experience sharp sudden pain and may suffer for several hours due to inflammation at the site of the procedure. This shock results in behavioural changes. The kids may shake their heads restlessly, scratch themselves or restrict their movement [100,101]. The heat generated by the tool can affect the brain, leading to convulsions, coordination problems and even death in extreme cases [100,102,103].

Dehorning may result in postoperative complications such as swelling, bleeding, infection or chronic discomfort. The healing process usually takes several weeks, which limits mobility and affects quality of life [98,104].

While pain can be reduced by the use of postoperative local anaesthesia and painkillers, these treatments are not completely effective [100,101]. Sedation or general anaesthesia are better options but require veterinary supervision and precise dosing to avoid complications [99,105].

Some alternative methods, such as clove oil injections, may cause less pain and reduce tissue damage, but they do not always effectively prevent further horn growth [106]. The safest approach is to combine local anaesthesia, sedation and pain medication, which significantly improves the welfare of goats during and after the procedure [100,101,107].

Another important procedure needed to maintain goat welfare is hoof trimming. If not performed regularly, goats are at risk of excessive hoof growth, as well as various diseases such as foot rot, interdigital dermatitis, white line disease, toe granuloma and deep joint sepsis; these represent the main causes of lameness, which leads to reduced feed intake, weight loss, reduced milk production and reproductive problems and may entail the premature culling of animals [108,109]. Regular hoof trimming helps prevent deformities and reduces the occurrence of toe granuloma and white line disease. It also helps maintain the correct hoof–pastern axis, which ensures that pressure is evenly distributed when standing and walking. This reduces chronic pain and the risk of lameness and promotes comfort of movement, normal activity and productive performance [16,110,111]. Although the procedure itself may cause short-term stress or discomfort, especially in the case of severely deformed hooves, a correctly performed correction minimises pain and allows the goat to quickly return to normal functioning [109,112].

Male goats may also undergo castration. Regardless of its purpose, castration constitutes a significant intervention in the body of the goat and has a direct impact on their welfare. Data indicate that the choice of method is associated with varying levels of pain. Hempstead et al. [111] indicate that surgical techniques are associated with both short-term and prolonged pain, which require appropriate preparation and post-operative support. The degree of comfort is also influenced by the age at which castration is performed: younger kids often undergo a milder procedure and recover more quickly, reducing the risk of complications and prolonged healing [113]. However, castration is sometimes delayed by producers as, despite the greater stress associated with the procedure, the presence of higher androgen levels may positively influence certain production traits, including muscle development [114]. It should also be emphasised that the choice of castration method has practical implications: mechanical techniques are popular and uncomplicated but also cause pain, while surgical castration carries the risk of more severe discomfort [111]. Therefore, the welfare of goats during castration depends on decisions regarding both the timing of the procedure and the technique used as well as the quality of care.

## 8. Specific Welfare Indicators for Goats

When assessing welfare in goats, there is a need for appropriate indicators. These should be easy to use, reliable and practical for everyday observation. Most of these indicators are non-invasive, with the key advantage of enabling a reliable assessment of animal welfare without imposing additional stress from capture, biological sampling, or prolonged handling. This approach is consistent with the AWIN, Anzuino, and Muri protocols, which were designed for routine use in farm settings. Most of the indicators used focus on negative aspects of welfare. In production settings, they facilitate the identification of health, nutritional, and environmental issues that require immediate intervention [11,13,16,17,19,115].

Currently, Qualitative Behaviour Assessment (QBA) is one of the few methods that systematically captures the qualitative dimension of herd behaviour, with inter-observer reliability confirmed. This method provides a valuable complement to quantitative indicators but cannot replace them for assessing health and environmental problems [116,117].

A complete set of goat welfare indicators, together with methods for assessing and interpreting the results, covers its physical, behavioural and environmental aspects (Table 6). Thus, it enables a comprehensive non-invasive assessment of animal health and welfare on the farm.

### 8.1. Body Condition Score (BCS)

Body condition score (BCS) is a reliable indicator of goat welfare, allowing for the assessment of fat and muscle reserves, which are crucial for health, milk production, reproduction and overall well-being [13,14,15]. The BCS evaluation reflects a direct assessment of the nutritional status of animals, regardless of body weight or skeletal size. Several scoring systems have been developed for goats. One of them is a three-point visual scoring system (very thin: −1, normal: 0, fat: 1 point) [17], which can be used by inexperienced observers without a significant reduction in reliability [11]. Another scoring system is a five-point scale (emaciated: 1, thin: 2, average: 3, fat: 4, obese: 5) [118]. The optimal BCS for mating is 3.0–3.5, and goats below 2.5 may show reduced hormonal activity and lower fertilisation rates. The following BCS ranges are recommended according to sex and physiological state: rams in the breeding season 3.0–3.5, pregnant goats 3.0–3.5, lactating goats 2.5–3.0 [119]. BCS is an indicator of animal welfare for which both intra-observer and inter-observer reliability have been confirmed [13,120].

### 8.2. Hair Coat Condition

The condition of the coat is also a good indicator of health and nutrition status. A dull, matted, flaky or excessively long coat may indicate nutritional deficiencies, the presence of parasites or chronic diseases. The assessment can be carried out visually from outside the pen and should cover the entire body, except for the head and limbs below the joints. Smooth, shiny and close-fitting coats are considered indictors of good condition, while dull and rough coats indicate poor welfare. The assessment is not carried out during the moulting period [17,18].

Research in Italy and Portugal by Battini et al. [60] found goats with dull, rough or matted coats to demonstrate significantly poorer body condition compared to goats with smooth and shiny coats; they were also characterised by a higher likelihood of mineral deficiencies (including magnesium, iron, potassium and calcium) and a higher incidence of abnormal lung sounds (e.g., wheezing, crackling, rales). The indicator demonstrated strong agreement between observers, confirming that it is reliable and can be successfully used in farm welfare assessment protocols.

### 8.3. Thermal Stress

Ambient temperature and humidity have a significant impact on the health and comfort of goats. Although goats are generally considered to be hardy and well adapted to harsh conditions, excessively high temperatures can lead to a decrease in appetite and performance due to heat stress. Conversely, low ambient temperatures in combination with wind or precipitation (THI < 55) may result in rapid body heat loss, thereby adversely affecting goat welfare [12,17,121].

Goats exposed to temperatures above 38 °C and a THI index of 75 show changes in behaviour, hormonal responses and metabolic adaptations, which are aimed at maintaining their thermal balance. These processes consume a significant amount of energy, drawing it away from growth and milk production [23,24].

Heat stress can be easily identified through observation. When overheated, goats breathe faster, pant with their mouths open and may drool excessively; in contrast, when experiencing cold stress, their coat becomes bristly, their body may be tense, they adopt a crouching posture and, in extreme cases, may shiver [20,121].

The number of goats showing signs of thermal stress, *viz.* overheating or hypothermia, is a valuable indicator of their welfare. Animals in comfortable conditions breathe calmly, keep their mouths closed and have smooth coats. Any deviation from the norm indicates that the thermal conditions in the goat housing facility require improvement [17].

### 8.4. Lameness

Lameness is an important indicator of pain and mobility problems. However, it is difficult to detect under typical farming conditions, and most protocols focus on identifying only the most severe cases [12,18,115]. A typical assessment is based on observing the gait, with any deviation from a normal gait being indicative of lameness: walking in a “goose-like” manner with straight limbs, walking on the knees, intense head shaking, and a pronounced curvature of the spine in the buttocks area [12,18,115]. Such observations should be carried out slowly, encouraging lying animals to stand up and take a few steps around the pen [18]. A goat exhibiting at least one of the above characteristics is classified as demonstrating severe lameness, while normal movement or minor deviations from normal are not classified [18].

### 8.5. Overgrown Claws

Hoof overgrowth is a common problem in dairy goat farming, with a prevalence ranging from about one-third [88] to nearly 80% of the herd [16]. This can lead to deformities [122] and lameness [16,122]. When the hooves do not wear down naturally (rearing on straw bedding, limited access to pasture) [123,124] or are trimmed too infrequently [16], the goat may experience excessive tissue growth and deformation.

Assessment involves a visual inspection of hoof shape, focussing mainly on the rear hooves: a hoof without a typical triangular profile is considered overgrown. In practice, based on the results, hooves can be classified as healthy, moderately overgrown (triangular profile preserved), or completely overgrown and deformed. A single severely overgrown hoof is enough to classify a goat as requiring attention [17].

The literature describes various methods of hoof assessment, ranging from lifting the limbs and scoring them on a scale of several degrees [19] to a simpler differentiation between moderately and severely overgrown hooves [16]. The AWIN protocol [18], however, recommends a binary assessment of the rear hoof as acceptable or unacceptable.

Photograph-based hoof assessment has also been found to be reliable. The method uses a three-point scale which takes into account the length of the toes, the shape of the heel and the shape and spacing of the hooves; in addition, the coronary joint is also assessed based on two categories. Although this method was found to achieve accurate results, it requires trained assessors and further validation on live animals [125].

### 8.6. Abscesses

Bacterial infections can result in the formation of external abscesses, i.e., localised thickenings of tissues filled with purulent contents; *Corynebacterium pseudotuberculosis* in particular can cause serious lymph node abscesses [126]. Such structures can appear on superficial lymph nodes as well as around wounds after injections. The disease causes significant economic impact on the small ruminant industry and has negative implications for animal welfare due to prolonged swelling and pain [17,127].

Abscesses can be detected visually during general observation of the herd without entering the pen. The most convenient time is during feeding, when the goats line up at the trough. The assessor walks along the feeder, observing the heads, necks and shoulders from a distance of 1.5 m, paying attention to any thickening and the presence of abscesses [17].

More thorough individual assessments can be performed by two assessors working together, with one in front of the animal and one behind. The first assesses the head and neck, and the second the rear part of the body, including the rump and udder. The sides of the body are usually omitted as they are not clearly visible [17,128].

During herd assessment, the number of goats with visible abscesses is recorded. The scoring does not distinguish whether the abscess has ruptured or whether scars are present. When assessing individual animals, a simple scale is used: no abscesses (score 0) or their presence (score 1). Even a single abscess in any part of the body results in the animal being assigned a score of 1 [17,60].

### 8.7. Qualitative Behaviour Assessment (QBA)

This method provides an insight into the emotional state of goats by observing their movement, posture and interaction within the group. Instead of assessing individual animals, the entire herd is analysed, paying attention to various signals, such as body tension, activity, exploration, aggression or signs of relaxation. Observations are made from locations that allow good visibility of all animals and should be performed when the goats are active and no earlier than 30 min after feeding. At the end of the session, all traits are scored on a visual analogue scale indicating their overall intensity in the group.

**Table 6:** Specific indicators of goat welfare.

Indicator	Assessment Method	Interpretation/Significance	Source
**BCS—body condition score**	Scale 1–5, palpation and visual assessment of fat and muscle reserves	1 emaciation, 2.5–3 optimal, ≥4 obesity: affects health, milk production and reproduction	[119]
**Condition of hair cover**	Visual assessment of the coat over the entire body (except the head and limbs)	Smooth, shiny—good condition; dull, tangled, flaky—health or nutritional problems	[17,60]
**Queues at the feeder**	Observation from outside the pen a few minutes after feeding	Long queues—limited access to feed, stress, conflicts within the herd	[13,17,57,60]
**Queues at the drinking trough**	15 min observation, counting goats standing in line (distance ~50 cm)	No queues—proper access; queues—stress and social tension; recommended max. 15 goats/drinking trough	[17,58]
**Bedding**	Visual assessment of thickness, softness, dryness and cleanliness of bedding material	Poor quality—reduced comfort, stress, reduced lying time and grooming behaviour	[17,44,46]
**Cleanliness**	Visual assessment of coat or bedding condition	Presence of dirt—hygiene and health issues	[15,16,17]
**Temperature stress**	Observing animals for signs of overheating or hypothermia	Overheating: rapid breathing, panting, drooling; hypothermia: bristled coat, crouched posture, trembling	[17,20,121]
**Kneeling by the feeder**	Observation of kneeling position at the feeding trough	Kneeling—uncomfortable feeder or difficult access to feed	[14,17,56]
**Lameness**	Observation of gait, nodding of the head, curvature of the spine	Presence of ≥1 feature—severe lameness, causes pain and affects mobility	[12,18,115]
**Overgrown claws**	Visual assessment of the rear hoof (triangular profile)	Moderately or completely overgrown—risk of deformity and lameness	[16,17,125]
**Abscesses**	Visual assessment of the head, neck, shoulders and rump during feeding	0—absent, 1—present; indicates bacterial infections	[11,17,18,60]
**Qualitative Behaviour Assessment (QBA)**	Observation of the entire herd, scoring traits on a visual analogue scale	Analysis of the emotional state of the herd (relaxation, curiosity, tension, aggression)	[17,117]

## 9. Protocols for Assessing Welfare in Goats

A number of goat welfare assessment protocols are in use. While they share a number of characteristics and are based on both animal characteristics and environmental conditions, they differ in various respects. The AWIN [17] and Anzuino [16] protocols employ both group- and individual-level assessments, while the Leite protocol [128] clearly distinguishes between animal-based and resource-based indicators. The Muri protocol [19] focuses exclusively on group or individual observation, covering selected physiological and behavioural indicators.

All four protocols take into account basic indicators such as lameness, body condition score, abscesses/swellings, cleanliness/faecal soiling and presence of discharges. In addition, the AWIN and Anzuino protocols also include a Qualitative Behavioural Assessment (QBA) and human–animal relationship assessment to evaluate behavioural and emotional aspects.

Some indicators are specific to certain protocols. The AWIN protocol additionally includes inter alia improperly regrown horns (improper disbudding), kneeling at the feeder (kneeling), queuing at the feeder and drinker (queuing at feeding/drinking), hair coat condition (hair coat condition), oblivion and thermal stress. The Leite protocol, on the other hand, places great emphasis on environmental indicators such as food and water availability, access to shade, herd density, cleanliness of equipment and type of bedding; while these are important for welfare, they are not discussed in detail in the animal assessment.

### 9.1. AWIN Protocol

The AWIN protocol is used to assess the welfare of adult dairy goats on intensive and semi-intensive farms. Its main aim is to evaluate animal health, care and natural behaviour without the use of invasive methods [18,123].

The method is based on a combination of direct observations and simple physical and behavioural indicators. The assessment is carried out in two stages. First, an initial quick check of the entire herd is performed, paying attention to easily verifiable indicators such as coat condition, leg cleanliness and the presence of abscesses [12,115]. If problems are detected during this general assessment, or if the farm as a whole is in the lowest performance group, a detailed analysis of individual animals is carried out. The second stage includes an examination of additional indicators such as udder asymmetry, hoof condition and individual behaviour [12,116], which allows for the identification of problems that may not be visible at the herd level.

The protocol is non-invasive and relatively quick: studies in Europe have found the entire assessment to take between one-and-a-half and two-and-a-half hours [12,115]. The AWIN offers the advantages of combining behavioural observation with physical assessment and being simple enough to allow different researchers to achieve similar results [13]. However, although it may be effective on intensive farms, it is not suitable for extensively reared goats or for young animals and billy goats [18,129]. Nevertheless, the protocol allows for the welfare of the entire herd and individual animals to be checked in a relatively short time, minimising stress to the animals and the workload for the assessor [15].

The AWIN protocol for assessing goat welfare is a practical tool; however, its application has several limitations. The protocol has been validated primarily in European breeds, and its reliability in other breeds or extensively managed systems remains uncertain. Some indicators, such as minor injuries, subtle behavioural changes, or early health problems, may be overlooked due to the focus on easily observable traits [11,130]. Behavioural observations, including Qualitative Behaviour Assessment, require trained observers, and short observation periods may not capture all relevant behaviours [14]. Therefore, the authors [14] point out that extensive training is necessary to properly assess animals using the QBA method. Additionally, certain assessments, such as body condition scoring in unhandled animals, may induce stress or be difficult to perform. Therefore, while AWIN provides valuable information, results should be interpreted with consideration of these constraints.

### 9.2. Anzuino Protocol

This protocol allows for the health and behaviour of adult dairy goats to be checked without disrupting farm operations, such as milking. The test consists of three stages: group assessment in pens, detailed examination of selected individuals in the milking parlour, and assessment of lameness when leaving the parlour. The test comprises body condition, udder and teat health, hoof condition, skin changes, cleanliness and behaviours indicating discomfort. The results provide reliable information that can support farmers and veterinarians in improving animal welfare [16]. Since the indicators strongly resemble those of the AWIN protocol, similar inter-observer reliability can be assumed [16].

A limitation of the Anzuino protocol is that it was developed for dairy goats, which may reduce its applicability to other breeds or production systems. The protocol also does not take into account the emotional state of the animals, which limits its ability to provide a comprehensive assessment of welfare [11]. Moreover, applying the protocol is time-consuming (approximately 24 h) and requires a milking parlour to observe individual animals [16].

### 9.3. Muri Protocol

The Muri protocol allows for a comprehensive assessment of dairy goat welfare based on a combination of animal observations and an analysis of husbandry conditions [19]. Its main purpose is to detect welfare problems and assess challenges related to the production system.

The assessment is carried out in two stages. First, the behaviour of the entire group of animals is observed using the QBA, which assesses the welfare of the herd at the group level. Following this, 20 individuals are randomly selected for a detailed individual analysis, including health status and physical characteristics [19].

The protocol also takes into account the quality of animal care by assessing the behaviour and approach of staff; these have a significant impact on the comfort and reactions of goats. The most common health problems identified are skin lesions, eye discharge, udder asymmetry, joint thickening and hoof overgrowth. In some herds, the results are more likely to indicate increased levels of anxiety [19].

Studies have found animal health and quality of care to have a significant influence on their behaviour and the type of flooring in the goat house to affect selected welfare indicators. Some indicators, particularly behavioural and emotional measures, require trained observers and can be influenced by subjective interpretation, potentially affecting inter-observer reliability [11]. The authors [19] emphasise that the protocol requires further validation, including the establishment of intervention thresholds tailored to specific farms. Despite these limitations, the protocol is a practical tool for detecting welfare problems and can be used as a basis for developing more comprehensive methods for assessing goat welfare [19].

### 9.4. Leite Protocol

The Leite protocol comprises a set of indicators developed to assess the welfare of meat goats in Brazil [128]. The protocol takes into account both animal characteristics and environmental conditions, including the hygiene of breeding facilities. The indicators can be used in semi-intensive and intensive systems, but their practical effectiveness requires further research.

The protocol includes 18 indicators based on two AWIN protocols (AWIN Goat, for dairy goats, and AWIN Sheep, for sheep) and one new indicator specific to local conditions (“cleanliness of facilities”). The indicators are divided into two main categories: animal-based indicators, which directly assess the physical condition and behaviour of animals and are the most reliable, and resource-based indicators, which assess environmental conditions and management.

Animal-based indicators include coat condition, apathy, lameness, approach test by a familiar person, and eye discharge; in addition, in intensive systems, these are accompanied by abscesses, body wounds, leg injuries, heat stress, soiling of the anal area and QBA. Resource-based indicators include water availability, access to shade or shelter, animal density, and cleanliness of housing [128].

A limitation of this protocol is the lack of validation regarding the indicators’ validity, reliability, and feasibility for meat goats. In addition, it was developed for specific local environmental conditions (Brazil) [128].

Table 7 provides an overview of the physical, environmental, behavioural and emotional aspects of goats, showing which indicators are common to all protocols and which are specific to a given system. The table is intended as a practical tool for comparing welfare assessment methods and selecting appropriate indicators depending on the type of husbandry.

## 10. Summary

Providing goats with adequate space, access to a varied environment and a diverse diet benefits their physical health, cognitive activity and natural behaviour while reducing stress and aggression. Furthermore, fostering positive social relationships, supporting the mother–kid bond and encouraging early contact with humans have significant positive influences on their emotional and social development.

The use of less invasive breeding practices, anaesthesia and pain-minimising measures also significantly improves welfare and reduces the risk of anxious behaviour by goats toward humans. These should be supported by systematic welfare assessments based on accepted protocols to allow for effective herd monitoring and the implementation of measures to improve quality of life. Furthermore, enriching the environment with physical, sensory and cognitive elements addresses the natural needs of goats more comprehensively.

However, achieving a high standard of goat welfare requires a holistic approach that includes optimising environmental conditions, humane husbandry practices, active environmental enrichment and regular assessment of the condition of the herd.

## Figures and Tables

**Table 3:** Comparison of goat feeding in natural and husbandry conditions and its impact on welfare.

Characteristic	Natural Conditions	Extensive Housing	Intensive Housing	Influence on Well-Being	Source
Type of feed	Leaves, shoots, grass, various plants	Mostly grass and leaves available in the pasture, partial selection	Mainly grass or mixtures; they do not have access to leaves, shoots, twigs and other parts of plants that they naturally eat	Limited food choice in intensive systems; in extensive systems, the animals can follow their natural feeding preferences, which increases behavioural satisfaction.	[8,51]
Feeder height and posture during feeding	Feeding at various heights, including trees (up to 2 m) Adopting different body positions while feeding	Mostly near the ground, sometimes leaves of shrubs up to 1–1.5 m	Most often horizontal. Feed administered on the floor or in simple troughs	Restricting foraging at different heights in intensive systems reduces natural control over the environment and behavioural expression. The inability to adopt natural feeding positions limits the expression of browsing and cognitive behaviours. In extensive systems, goats can partially express their natural postures and activity. The possibility of foraging near bushes and low trees supports natural exploratory behaviour.	[8,50,51,52,53]
Feed selection	High—possibility of choosing preferred plants	Moderate—animals can choose from available vegetation	Low—homogeneous mixtures, no selection	Food selection is limited in intensive systems, which reduces motivation and feeding satisfaction; in extensive farming, goats can choose from available plants, thus supporting their natural preferences.	[8,51]
Feeding time	Varies depending on the time of day and season	Relatively natural depending on pasture availability	Often fixed, less variable	In intensive systems, feeding times are set artificially, which restricts natural rhythms and behavioural activity; in extensive systems, goats maintain more natural activity patterns.	[51]
Potential for exploration	Large—can search the area, observe the surroundings	Moderate—can search pasture	Restricted to housing space	Limited cognitive stimulation in intensive systems can lead to stress and boredom; in extensive systems, the animals have greater opportunities for exploration, which supports their mental well-being.	[8,54]

**Table 4:** Types of environmental enrichment for goats and their impact on welfare.

Form of Enrichment	Elements	Impact	Sources
Physical/structural	Access to outdoor enclosure and branches	Increased activity and exploration, observation of outdoor play, initially reduced rest time, tendency towards more aggressive interactions	[54]
	Scratching brushes	Stimulation of care, exploration, stimulation of touch	[68]
	Wooden platforms/climbing platforms	A place to relax, climb, play, more usable space, physical activity	[68]
	Climbing trunks, tyres, PET bottles with corn	Greater activity, adopting natural body positions, reducing stereotypical behaviour	[66]
	Boxes, tyres	Exploration, interaction with the environment, reduction in repetitive behaviours	[69]
	Structural and cognitive connection	Better learning abilities, greater willingness to explore new environments	[63]
Sensorial	Music, sound	Stimulation of the senses, stress reduction	[63]
Social	Positive interactions with humans	Stress reduction, improvement of animal–carer relationships	[63]
Cognitive	Training devices that reward with food or water	Cognitive development, improved learning, increased exploration	[63]

**Table 5:** Tests evaluating the reactions of goats to human contact and handling.

Test	Procedure	Measured Parameters	Interpretation	References
Reactions to a motionless person in a test environment (RSH-T)	Goat left in the test area for 5 min, looks though a fence, man stands motionless	Time spent in contact with humans, number of vocalisations, heart rate	Reaction to human presence, stress level	[95]
Reactions to a moving person in a test environment, person moves their hand (RMH-T; MoveHd)	Goat left alone in the test area for 1 min, a person enters and stands still for 1.5 min, then tries to stroke it for 1.5 min	Time spent near humans (<2 m), contact, vocalisations, sections covered	Degree of positive reaction to human contact	[88]
Reactions to a stationary person in a home environment and to a moving person (RSH-H; RMH-H App—person approaching)	Three parts, 2–3 min each: a man stands still, man walks along fence, man tries to touch goat	Approach latency (<1 m), contact, time spent near humans	The level of curiosity and comfort towards humans	[93,94]
Reactions to a moving person in a test environment, person approaching (RMH-T App, treadmill)	The goat moves on a circular treadmill, the human follows at 0.5 steps/second for 3.5 min; blood sampling before and after	Escape distance, following, approaching, avoidance, vocalisation, contact with humans	Level of fear and acceptance of contact with humans; physiological stress	[97]
Reactions to manipulation/handling in the housing environment (RHd-H)	Milking twice a day for 21 days; behaviour assessed by observer	Milk production and seven behavioural scales: 1. excitability, 2. tension, 3. alertness, 4. anxiety, 5. confidence, 6. friendliness towards humans, 7. fear of humans	Assessment of sustained reactions to human contact and handling; comfort and stress levels	[93]

**Table 7:** Goat welfare assessment protocols.

Protocol	Group	Level of Assessment	Type of Indicators	Domain and Indicators	Procedure	Sources
**AWIN**	Adult dairy goats	Group and individual	Animal-based	**Nutrition:** BCS, access to water; **Environment:** cleanliness, thermal stress, bedding; **Health:** lameness, swellings, overgrown claws, udder abnormality, discharges, improper disbudding, dyspnoea, coughing; **Behaviour:** kneeling, queuing at feeding and drinking points, Qualitative Behaviour Assessment (QBA), human–animal relationship (HAR), oblivion; **Mental state:** anxiety, emotional well-being.	Non-invasive, two-stage assessment (rapid herd screening—detailed individual analysis)	[12,13,17,18,60,115,116,123]
**Anzuino**	Adult dairy goats	Group and individual	Animal-based	**Nutrition:** BCS, access to water; **Environment:** cleanliness, thermal stress; **Health:** lameness, swellings, overgrown claws, udder abnormality, lesions; **Behaviour:** kneeling; oral behaviour.	Non-invasive, three-stage assessment: group assessment, detailed examination of selected individuals, assessment of lameness upon leaving the hall	[16]
**Muri**	Adult dairy goats	Group and individual	Animal-based	**Nutrition:** BCS; **Environment:** cleanliness; **Health:** lameness, swellings, overgrown claws, udder abnormality, lesions, improper disbudding, discharges, chest girth; **Behaviour:** agonistic interactions, QBA, HAR; mental state: anxiety level.	Non-invasive, two-stage assessment: group behaviour—20 individuals selected at random; includes quality of care and rearing conditions	[19]
**Leite**	Meat goats	Group and individual	Animal-based + Resource-based	**Nutrition:** BCS, access to water; **Environment:** cleanliness of facilities, thermal stress, access to shade and shelter, stocking density; **Health:** lameness, swellings, lesions, improper disbudding, discharges, leg injuries; **Behaviour:** QBA, HAR, oblivion; **Mental state:** apathy, heat stress.	Non-invasive, takes into account both animal welfare and environmental conditions; AWIN protocol adapted for local conditions in Brazil. Based on indicators used in AWIN protocol plus indicator: “cleanliness of facilities”	[128]

## Data Availability

No new data were created or analyzed in this study. Data sharing is not applicable to this article.
